# Tuberculosis in Newborns: The Lessons of the “Lübeck Disaster” (1929–1933)

**DOI:** 10.1371/journal.ppat.1005271

**Published:** 2016-01-21

**Authors:** Gregory J. Fox, Marianna Orlova, Erwin Schurr

**Affiliations:** 1 McGill International TB Centre, McGill University, Montreal, Quebec, Canada; 2 Respiratory Epidemiology and Clinical Research Unit, Montreal Chest Institute, Montreal, Quebec, Canada; 3 Program in Infectious Diseases and Immunity in Global Health, The Research Institute of the McGill University Health Centre, Montreal, Quebec, Canada; 4 Departments of Medicine and Human Genetics, McGill University, Quebec, Canada; Stony Brook University, UNITED STATES

## Abstract

In an accident later known as the Lübeck disaster, 251 neonates were orally given three doses of the new Bacille Calmette–Guérin (BCG) antituberculosis (TB) vaccine contaminated with *Mycobacterium tuberculosis*. A total of 173 infants developed clinical or radiological signs of TB but survived the infection, while 72 died from TB. While some blamed the accident on BCG itself by postulating reversion to full virulence, such a possibility was conclusively disproven. Rather, by combining clinical, microbiological, and epidemiological data, the chief public health investigator Dr. A. Moegling concluded that the BCG vaccine had been contaminated with variable amounts of fully virulent *M*. *tuberculosis*. Here, we summarize the conclusions drawn by Moegling and point out three lessons that can be learned. First, while mortality was high (approximately 29%), the majority of neonates inoculated with *M*. *tuberculosis* eventually overcame TB disease. This shows the high constitutional resistance of humans to the bacillus. Second, four semiquantitative levels of contamination were deduced by Moegling from the available data. While at low levels of *M*. *tuberculosis* there was a large spread of clinical phenotypes reflecting a good degree of innate resistance to TB, at the highest dose, the majority of neonates were highly susceptible to TB. This shows the dominating role of dose for innate resistance to TB. Third, two infants inoculated with the lowest dose nevertheless died of TB, and their median time from inoculation to death was substantially shorter than for those who died after inoculation with higher doses. This suggests that infants who developed disease after low dose inoculation are those who are most susceptible to the disease. We discuss some implications of these lessons for current study of genetic susceptibility to TB.

## Author Summary

The Lübeck disaster is a unique event in the history of tuberculosis when 251 newborns were accidentally infected with a virulent strain of *Mycobacterium tuberculosis*. The disaster happened while BCG was introduced as an anti-TB vaccine. In an exemplary multidisciplinary investigation, the disaster was shown to be due to the accidental contamination of BCG vaccine preparations with virulent *M*. *tuberculosi*s and not BCG itself. A meticulous description of the accident, including epidemiological, histopathological, and bacteriological observations, was published; however, this report is only available in German, thus limiting access to the data. Here, we present a summary of the accident and its clinical consequences. Based on the results of the initial report, we draw three important conclusions: (i) overall, the newborns demonstrated remarkable resistance to virulent *M*. *tuberculosis*; (ii) the extent of contamination had a strong impact on the observed TB clinical spectrum, demonstrating the importance of the infectious dose for innate resistance to TB; and (iii) the possible effect of host genetics on TB susceptibility appeared most prominent in the context of a low dose of *M*. *tuberculosis*. The latter observation suggests that low exposure settings may be best suited for host genetics studies of susceptibility to TB.

## Introduction

Tuberculosis (TB) is an enduring global public health challenge, with 9 million new cases of disease each year [1]. Approximately one-third of the world’s population has been infected with *Mycobacterium tuberculosis*, yet less than 10% of infected individuals develop clinical disease during their lifetimes [2]. Understanding why only some individuals exposed to the bacilli develop disease is of paramount public health importance and has been a major focus of global TB research efforts [3]. A range of intrinsic biological factors contribute to an individual’s risk of developing disease. Among these, human genetics studies have identified some common variants that confer a modest risk of disease and some rare variants that can determine severe forms of TB [4]. However, for most infected people who develop disease, a predisposing or determining factor is still lacking. Most importantly, the impact of exposure length and intensity, i.e., the infectious dose, on the risk of developing clinical TB is unknown.

*M*. *tuberculosis* is typically transmitted via the respiratory route. The bacilli are deposited by aerosol in the lung alveoli, where they are taken up by alveolar macrophages, interstitial lung macrophages, dendritic cells, and neutrophils. Dendritic cells provide a major conduit for the transport of *M*. *tuberculosis* to the lymphatic system. If innate immunity effector cells fail to control the infection in the lung, dendritic cells will migrate to the regional lymph node and present *M*. *tuberculosis* antigens to T cells [5]. This will trigger an acquired immune response by which antigen-specific T cells migrate to the lung and secrete cytokines, such as IFN-γ, that enhance macrophage microbicidal activity. This can result in effective killing of all tubercle bacilli, the formation of infectious granulomas that wall off the bacilli and prevent further spread of the infection, or can prove ineffective, resulting in the development of primary TB disease that may spread from the lung to other organs (extrapulmonary, disseminated TB). The latter outcome is more typically found in pediatric TB, and very young children are generally considered to be extremely susceptible to TB disease [6,7]. A TB-like disease can also be caused by infection with *M*. *bovis*, which is typically transmitted by consumption of contaminated milk [8]. Because of the oral route of infection, the number of extrapulmonary granulomas is higher than in *M*. *tuberculosis* TB [9]. The cellular mechanisms of pathogenesis are thought to be independent of the route of infection, while tissue-based pathogenesis depends on the route of infection.

Accounts of the natural history of TB from the pre-antibiotic era can provide valuable insights into variation in disease susceptibility. The “Lübeck disaster,” unfolding in the spring of 1930, offers such insights. At that time, 251 newborn babies were accidentally inoculated with *M*. *tuberculosis*-contaminated Bacille Calmette–Guérin (BCG), an attenuated form of *M*. *bovis*, instead of a pure substrain of the live BCG vaccine. In the follow-up to the accident, the establishment of a uniform dataset and the systematic evaluation of the clinical and epidemiological data were done by Dr. Albert Moegling of the German Tuberculosis Research Institute and the Federal Public Health Office. Accurate details of the outbreak have only been available in German [10]. Here, we recapitulate the events and outcomes of the incident, based on Moegling’s meticulously recorded clinical and pathological data, and examine this outbreak from a fresh perspective, attempting to draw some lessons for our understanding of this challenging and devastating disease.

## Description of the Accident

Beginning in December 1929, the Lübeck General Hospital embarked on a campaign to prevent TB. All newborns were offered the newly available live BCG vaccine (strain 374) within the first ten days of life, although not all parents accepted vaccination. Unfortunately, vials of vaccine were inadvertently contaminated with live *M*. *tuberculosis* bacteria at unknown proportions. The source of contamination was an easily identifiable, uncommon strain of *M*. *tuberculosis* (Kiel strain) that was cultivated at the vaccine preparation laboratory. Over the next four months, 251 of the 412 newborn babies were given a contaminated vaccine.

For vaccine batch preparation, BCG was grown for 14 days on egg-nutrient agar, taken up in a glycerol–glucose solution, and adjusted to desired bacterial content by density determination. A total of three aliquots, each corresponding to 2 ml, were packaged for each infant. Most commonly, the vaccine stock for a given vaccination day was obtained from pools of two to four harvested agar cultures. For vaccine administration, vaccine vials were vigorously shaken, and re-suspended bacterial emulsions were added to a spoon of warm breast milk. Each dose contained the equivalent of 10 micrograms of a solid bacterial culture. The recommended administration of the vaccine involved three separate oral doses of the bacterial emulsion, to be given during the first ten days of life. Each dose was separated by two days from the next inoculation, implying that the first dose was received no later than day six of life. The vaccine was given by mothers, midwives, or hospital nursing staff using a medicine spoon. Occasionally, if infants did not swallow the vaccine, their noses were held until it was swallowed. Dr Moegling’s report indicated that all but 14 children received the prescribed three doses. Some children vomited or experienced diarrhea soon after ingestion and/or up to one week following vaccination. Following extensive interviews with caregivers, Moegling concluded that while there were deviations from the recommended protocol (e.g., too much liquid, heating of the preparation, the use of water or diluted milk instead of breast milk), such deviations were the exception and did not impact the main conclusions reached in his report.

Within the next three months, a substantial number of infants began to die, and a public health investigation was launched. Autoradiographs were conducted on a total of 228 children, while tuberculin skin tests (TST) were performed on 212 infants as part of their clinical assessments. Surviving children were followed closely until 1933—three years after the outbreak began—since no antibiotic therapy to treat the disease was yet available. Abdominal radiography was performed on surviving children in 1932 and 1933 to record the presence of mesenteric lymph node calcification, a sign of mesenteric TB. For deceased children, an autopsy was performed. Deaths were classified by pathologists as either being related to TB or due to another cause [11]. The socioeconomic status of each infant was also determined, on the basis of a questionnaire administered to their parents.

## Clinical Outcomes of Exposed Children

Among the 251 inoculated children, 228 (90.8%) developed some clinical or radiographic evidence of TB disease (Table 1), and 77 infants (30.7% of those inoculated) died within a year of inoculation. Of those who died, five were thought to have died of TB-unrelated causes, while the remaining 72 demonstrated clinical evidence of extensive TB. While the parents of four of the children refused permission for autopsy, pathology on autopsies of all remaining 68 children confirmed the clinical diagnosis of TB disease [11]. In contrast, of the 164 non-inoculated children born during the same period, 19 (11.6%) died before the age of three, including 16 before the age of one, from TB-unrelated causes. We calculated that mortality in the first year of life was 3.1 (95% CI 1.9–5.1) times higher in inoculated than non-inoculated children. Male children were more likely to die during follow-up than females (relative risk [RR] 1.6, 95% CI 1.0–2.3) (S1 Table). Of note, the proportion at year one of non-TB–related deaths in the inoculated children was significantly reduced compared to non-inoculated neonates (*p* = 0.002).

**Table 1:** Characteristics of vaccinated children.

Characteristic	Number	(%)
Total infants born	412	
Infants vaccinated	251	(60.9%)
Male gender, among vaccinated children	137	(54.6%)
Cases of TB, among vaccinated children	228	(90.8%)
Site of TB (*n* = 228)		
Lymph node	227	(99.6%)
Abdominal	197	(86.4%)
Oropharynx	182	(79.8%)
Pulmonary	30	(13.2%)
Ear	20	(8.8%)

**Fig 1:**
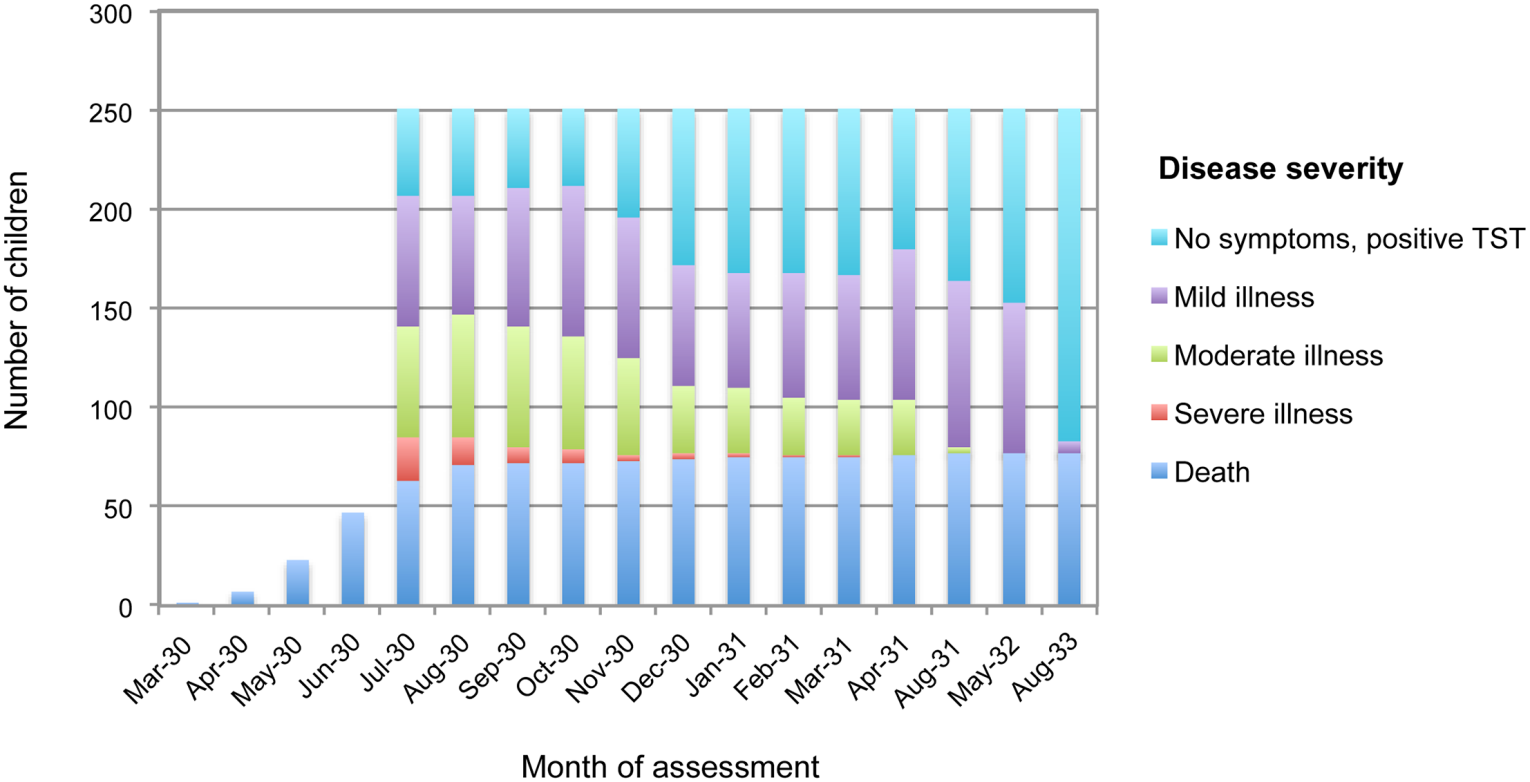
Summary of clinical status of TB in newborns during three-year follow-up. The figure uses data extracted from Tables 8 and 9 in Moegling’s report. All but six children who survived until September 1933 achieved complete clinical remission. Six of the seven clinical categories of TB are presented (excluding “no signs of disease and a negative TST”). Severe illness in this figure combines both (a) severe illness with worst prognosis and (b) severe illness with questionable, eventually unfavourable, prognosis.

Since the prognosis for *M*. *tuberculosis*-exposed children was the dominating concern at the time of the accident, TB disease was classified by the investigating team in one of seven categories in order of improving prognosis: (1) death; (2) severe illness with worst prognosis; (3) severe illness with questionable, eventually unfavourable, prognosis; (4) moderately ill with questionable, eventually favourable, prognosis; (5) mild illness with clear clinical symptoms of TB, yet a good prognosis; (6) no clearly detectable signs of TB, but with positive TST; (7) no signs of disease and a negative TST. A total of 156 infants developed TB yet recovered spontaneously from the disease in the absence of effective treatment. The clinical history of TB in inoculated children is shown in Fig 1. Only six surviving children demonstrated ongoing signs or symptoms of active disease after three years. Follow-up investigations in subsequent years detected autoradiographic evidence of disease in 17 apparently healthy children.

The relationship between the time of inoculation and outcomes was carefully evaluated. Moegling observed that the proportion of children with severe disease varied markedly according to the day on which they had been inoculated. For example, among 23 newborns inoculated between March 25 and 27, 1930, 17 (74%) died and four (17%) developed severe TB. In contrast, of 20 children inoculated between April 1 and 5, 1930, none (0%) died of TB-related causes and 11 infants (55%) developed mild disease. Understanding the pronounced differences in outcome for different vaccination days was a major question in the investigation of the outbreak.

As a first step, Moegling considered different operational and demographic causes of the observed heterogeneity in outcome, including a possible role of specific caregivers, sex of children, family history of TB, socioeconomic status of parents, vaccine preparation, adverse effects, and deviations from feeding protocol such as administration of less than the three required doses of the BCG preparation. While some of these factors had an impact on individual outcome, none could explain group outcomes for specific vaccination days. Specifically, breast milk had no discernible impact on either mortality or severity of TB. Likewise, secondary childhood infections were not substantial confounders of severity of TB. Hence, Moegling postulated that group outcome heterogeneity reflected contamination of the BCG vaccine preparations with differing amounts of *M*. *tuberculosis* for each vaccination day. He allocated infants to one of four strata (called “virulence strata” 1–4), defined according to the proportion of children who died or developed severe disease with poor prognosis on the day that the first dose was administered. The method by which cutoffs for these strata were chosen was not reported in detail. A quantitative determination of contamination levels in individual vaccine batches given to infants was not possible. One of the treated children was assumed to receive a pure BCG vaccine called “virulence stratum 1” (*n* = 1). Stratum 2 (*n* = 93), stratum 3 (*n* = 83), and stratum 4 (*n* = 74) were classified in order of increasing mortality based on the day of administration, and stratum was therefore associated with group mortality. Increased group mortality was attributed to an increased dose of *M*. *tuberculosis* in the vaccine batch. The clinical outcomes of infants in each of Moegling’s strata are shown in Table 2.

**Table 2:** Outcomes and time to onset of TB symptoms and death, grouped by “virulence stratum” as defined by Moegling.

	Virulence stratum^a^			
Characteristic	2 (Lowest)	3	4 (Highest)	Overall
Total, *n*	93	83	74	250^b^
Peak clinical severity of disease^c^				
Death from TB (*n*, %)	2 (2.2%)	17 (20.5%)	53 (71.6%)	72 (28.8%)
Severe TB (*n*, %)	9 (9.7%)	34 (41.0%)	18 (24.3%)	62 (24.8%)
Moderate TB (*n*, %)	63 (67.7%)	29 (35.0%)	3 (4.1%)	94 (37.6%)
No clinical signs of TB, positive TST (*n*, %)^d^	15 (16.1%)	2 (2.4%)	0 (0.0%)	17 (6.8%)
Died of unrelated causes^e^	4 (4.4%)	1 (1.2%)	0 (0.0%)	5 (2.0%)
Days to onset of clinical TB after inoculation among deceased children (median, interquartile range [IQR])^f^	42 (35–49)	35 (28–63)	49 (35–56)	42 (28–56)
Days to death from TB after inoculation (median, IQR)^g^	59 (57–61)^h^	93 (66–118)^i^	87 (70–111)	86 (67–114)
Odds of death compared to low virulence stratum (odds ratio [OR], 95% CI)	Reference	12 (6–25)	115 (54–246)	—

^**a**^ “Virulence stratum” represents the presumed level of contamination in the BCG administered. The virulence stratum was inferred by Moegling from the proportion of children dying depending on the day of inoculation.

^**b**^ One child, who was considered to receive pure BCG (group 7, virulence stratum 1), is excluded from this table.

^**c**^ Moegling’s report divided children according to one of seven final disease states: (1) death; (2) severe illness with worst prognosis; (3) severe illness with questionable, eventually unfavourable, prognosis; (4) moderately ill with questionable, eventually favourable, prognosis; (5) mild illness with clear clinical symptoms of TB, yet a good prognosis; (6) no clearly detectable signs of TB, but with positive TST; (7) no signs of disease and a negative TST (excluded here). “Death from TB” also includes initial group 2. Groups 3 and 4 are combined as “Severe disease.”

^**d**^ The 17 apparently healthy infants did show radiological signs of TB at two- or three-year follow-up.

^**e**^ On autopsy, five children were found to have died without evidence of tuberculosis.

^**f**^ The time of onset to clinical symptoms was recorded for 71 of 72 children who died of TB. The time to onset of clinical TB was not recorded for other children.

^**g**^ The time to death was calculated from the reported date of inoculation and date of death.

^**h**^ Using the Wilcoxon–Mann–Whitney test, the time to death after inoculation was not associated with falling in groups 3 or 4 versus group 2 (*p* = 0.12).

^**i**^ Includes data for 16 of 17 individuals, as the duration of follow-up was missing for one individual.

In the absence of convincing alternative hypotheses, Moegling’s explanation of different levels of contamination in the four virulence strata is supported by four observations: (i) Follow-up testing of two sources of BCG from the Lübeck laboratory revealed variable degrees of contamination with *M*. *tuberculosis*. First, analysis of BCG stock cultures, including those potentially used for vaccine preparation, revealed variable contamination with *M*. *tuberculosis* (Kiel strain) in five of 12 tested cultures. One of those cultures was revealed as pure *M*. *tuberculosis* [12]. In addition, leftovers from ten batches of used vaccine vials were put in culture, and, in three instances, mycobacteria could be grown. Follow-up studies in animal experiments showed that two cultures were made up of pure BCG, while from one batch, only *M*. *tuberculosis* could be cultivated [12]. (ii) The number and extent of infection foci detected either by autopsy or during roentgenographic follow-up of surviving children correlated strongly with the virulence strata. (iii) Among the 74 children given the vaccine with the highest level of *M*. *tuberculosis* contaminant—virulence stratum 4—three of the four children who missed at least one dose survived. The risk of death was 2.9 (95% CI 1.5–5.8) times higher among children who took all doses compared to those who missed one or more doses. (iv) Vomiting and diarrhea within a week of inoculation were associated with a substantial reduction in mortality. The relative risk of death with vomiting and diarrhea was 0.32 (95% CI 0.15–0.68) compared to those who did not vomit or develop diarrhea (S2 Table). Presence of primary foci in the lung of some of the children with vomiting strongly suggested that these children aspirated part of their vomitus. When taking into account 221 children without evidence for vomitus aspiration, the protective effect of diarrhea and vomiting was substantially stronger, with a relative risk of death of 0.06 (95% CI 0.01–0.46).

For the 72 infants who died of TB, the median time from inoculation to disease onset was 42 days (IQR 28–56 days), as shown in Table 2. The time from first inoculation to onset of disease was independent of the inferred level of contamination. The median time from inoculation to death was 86 days (IQR 67–114 days), with a range of 34 to 363 days. Death occurred more rapidly in the two individuals who died after being given a “low dose” of *M*. *tuberculosis* (virulence stratum 2), although this did not reach statistical significance (*p* = 0.12).

Children inoculated with preparations of the same vaccine batch displayed a spectrum of clinical symptoms ranging from death to very mild signs of TB. This observation is often quoted as evidence for variable innate, possibly genetically controlled, resistance to TB disease. However, mild and moderate disease followed the mortality classification scheme very well. For example, in the lowest virulence stratum, 16.1% of children showed no overt clinical symptoms of disease, while 67.7% displayed moderate TB. In contrast, none (0%) of the children in the highest virulence stratum were without any clinical symptoms, and only 4.1% displayed moderate TB disease (Table 2). Therefore, the proportions of neonates with mild or no symptoms were strongly correlated with deduced dose, suggesting that innate resistance can be overcome by increasing exposure intensity to *M*. *tuberculosis*.

## Key Lessons from the Lübeck Disaster

The Lübeck disaster was a tragic chapter in the history of TB control from which several valuable insights about susceptibility to TB can be drawn. A major concern at the time was that BCG itself had become virulent. Such a possibility was proven to be wrong based upon bacteriological, epidemiological, and clinical grounds. Since this outcome is widely known, we will point out some other less-quoted implications.

### 1. Newborn infants have a remarkable resistance to *M*. *tuberculosis*

In spite of the tragic deaths of 72 children from TB, a striking feature of the outbreak was the remarkable resilience of the remaining children who had been exposed to the mycobacterium. Careful clinical observation of the 174 surviving children found that only six children remained unwell by September 1933. Overall, 68% of those who had developed clinical disease achieved a spontaneous resolution of their symptoms. This is similar to findings in the limited number of other studies in children from the pre-antibiotic era [7]. It is worthwhile to keep in mind that the mode of infection in the accident more closely resembles *M*. *bovis*, which is commonly transmitted via the oral route.

### 2. Infectious dose as key determinant of outcome

The second lesson from the Lübeck disaster is the important relationship between dose and outcome. Clear differences were observed in the mortality rate according to the day of vaccine administration. Although the concentrations of virulent bacteria were not measured at the time of inoculation, temporal trends in prognosis suggested that an environmental factor(s) explained much of the variability in outcome between individuals. The chief investigator of the outbreak, Moegling, concluded that variation in dose of *M*. *tuberculosis* was the most likely explanation for the temporal variability. This explanation was directly supported by the bacteriologic workup that detected pure cultures of BCG, variable levels of *M*. *tuberculosis* in BCG cultures, and pure *M*. *tuberculosis* cultures. Moegling concluded that, as between two and four stock vials were used to prepare each vaccine batch throughout the outbreak, it was quite possible that the different batches used for inoculation of neonates contained *M*. *tuberculosis* at different concentrations. Likewise, as mentioned above, any deviation from the protocol that resulted in reduced uptake of inoculum had substantial beneficial effects for neonates.

While we cannot be certain that Moegling’s explanation is correct, as the concentration of the virulent bacteria was not directly measured, his explanation is consistent with all known facts of the disaster. As pointed out by Moegling, we must then conclude that with an increasing dose of *M*. *tuberculosis*, the role of any individual resistance (e.g., genetic resistance to disease) would be less important than the effect of the dose—i.e., the effect of a high level of exposure outweighed the natural resistance to the bacillus. In this context, the general shift of disease severity with increasing dose further supports the dominating role of the quantity of ingested *M*. *tuberculosis* for the clinical picture.

### 3. Children who died after receiving a low dose appear to have been more susceptible

The median time intervals from exposure to the occurrence of clinical symptoms were very similar irrespective of the infectious dose (Table 2). On the other hand, the time to death was particularly short among the two children exposed to a “low dose” (a median of 59 days for virulence stratum 2 compared to 86 days for the whole cohort) (Table 2). Although the numbers here are small, and, hence, do not reach statistical significance, this observation does raise some interesting possibilities. It is possible that these two individuals were particularly susceptible to the disease, given the rapid progression in the setting of a low “virulence dose.” In light of the observation that the quantum of exposure is likely to have a major effect on prognosis, it may be that the effects of individual susceptibility are best seen at the lower end of the dosing spectrum. Adapted to present-day conditions, this might imply that “sporadic” cases without known index case are more likely to reveal strong genetic susceptibility. This observation had been previously made in the context of interleukin-12 receptor β1 deficiency but also for pediatric TB and the impact of the *NRAMP1* gene on TB susceptibility [13,14].

## Implications for the Understanding of the “TB Susceptibility” Phenotype

The Lübeck disaster offers some valuable insights into the relationship between environmental exposure and innate susceptibility to TB. In particular, the importance of environmental exposure in disease progression could explain why human genetic studies of TB, to date, have failed to identify as many replicated susceptibility genes as in other complex diseases. For example, in contrast to leprosy (caused by *M*. *leprae*), for which a number of potential susceptibility candidate genes have been identified [15,16], results of genetic association studies in TB disease have identified relatively few loci, each with a very modest effect, that could be replicated consistently in different populations [17–19]. In leprosy, the mode of transmission is still unknown, but given the unusually long exposure times to cause disease, it seems likely that effective infectious doses are generally lower than in TB, which might account for the selection of strong genetic effects, especially among young, early-onset leprosy cases [20].

Despite its historical nature, important implications of the accident for the present-day study of TB susceptibility can be seen. In the present antibiotic era, TB patients are treated, and we do not know which of the patients would have self-healed. Hence, the pronounced shift away from no or moderate symptoms of TB (which might go unnoticed) toward more severe TB disease with increasing dose poses a huge challenge for phenotype definition of TB susceptibility. The data clearly suggest that TB susceptibility for molecular studies needs to be defined in the context of a given dose—something that has been accepted by researchers working on the mouse model for a long time. It appears the best approach to this challenge is to work with “sporadic” cases in low-transmission settings that, on average, are expected to have been exposed to a lower dose. In contrast, drawing inferences about dose in high transmission settings is more difficult. Low-dose sporadic patients likely also have stronger genetic susceptibilities, as pointed out above.

The tragic incident provides a unique window into the natural history of TB in the neonatal period. The vaccine administration dates were carefully documented by Moegling for those children who died, permitting the duration between exposure and onset of clinical symptoms to be determined. A median time of 42 days to onset of clinical disease and 86 days to death is consistent with the time span for TB onset in early childhood recorded in the pre-antibiotic era [7]. The regression of disease for the majority of neonates is consistent with early descriptions of so-called “self-cure” [7]. Furthermore, children with moderate disease had persistent signs and symptoms for up to a year before either being cured or succumbing to the infection.

## A Historical Event

Despite careful efforts, there were some limitations preventing investigators from getting definite answers about the accident. The major limitation was the absence of experimental quantification of the dose of *M*. *tuberculosis* that was administered to children. Furthermore, the precise details of the way in which the vaccines were drawn from the contaminated flasks were not documented. Despite these limitations, the available evidence supports the principal conclusion reached by the investigative team: children were inoculated with a BCG preparation that contained *M*. *tuberculosis*, and the quantum of *M*. *tuberculosis* contaminant was not constant but varied between different vaccine batches.

A further limitation in evaluation of temporal trends of clinical disease is the bias in the timing of diagnosis of children—diagnostic delay was more likely at the start of the vaccination campaign disease, before the outbreak was recognized. By the end, clinicians were more vigilant for early symptoms of disease and would have diagnosed the disease earlier, and clinical care might have been more focused in the later stages of the accident.

Finally, observations about the clinical course of TB in the inoculated children cannot necessarily be generalized to modern childhood TB, which is acquired by usual airborne transmission rather than by oral administration of a concentrated bolus of bacteria. In Lübeck, contaminated BCG was administered orally, resulting in a high proportion of oropharyngeal and gastrointestinal disease. It is likely that infection via the respiratory route would have resulted in more severe disease as is suggested by the increase of mortality in children with pulmonary foci as compared to those who did not display lung involvement. Nonetheless, lessons about timing from exposure to disease and death are likely to be broadly similar.

In conclusion, the Lübeck accident caused a major global scandal at the time, on account of the tragic circumstances. However, it has an enduring legacy in demonstrating the importance of exposure intensity to *M*. *tuberculosis* in determining outcomes of TB and highlighting the importance of accounting for environmental factors when examining host susceptibility to disease.

## Supporting Information

S1 Table The relationship between gender and death among inoculated children. (DOCX)

S2 Table The relationship between vomiting and/or diarrhea and death among 251 inoculated children. (DOCX)
