# Supplementary material for: Tuberculosis in Newborns: The Lessons of the “Lübeck Disaster” (1929–1933)
Source: PLoS Pathog. 2016 Jan 21;12(1):e1005271. doi: 10.1371/journal.ppat.1005271 (PMC4721647; doi:10.1371/journal.ppat.1005271)
Supplement: S1 Table — (DOCX) [file ppat.1005271.s001.docx]

**S1 Table:** The relationship between gender and death among inoculated children

| **Gender** | **Died (n=77)** | **Survived (n=174)** |
| --- | --- | --- |
| Male | 50 (36.5%) | 87 (63.5%) |
| Female | 27 (24%) | 87 (76%) |
