# Supplementary material for: Tuberculosis in Newborns: The Lessons of the “Lübeck Disaster” (1929–1933)
Source: PLoS Pathog. 2016 Jan 21;12(1):e1005271. doi: 10.1371/journal.ppat.1005271 (PMC4721647; doi:10.1371/journal.ppat.1005271)
Supplement: S2 Table — (DOCX) [file ppat.1005271.s002.docx]

**S2 Table:** The relationship between vomiting/diarrhea and death among 251 inoculated children

| **Vomiting/Diarrhea** | **Died (n=77)** | **Survived (n=174)** |
| --- | --- | --- |
| Vomiting and diarrhea | 7 (12.5%) | 49 (87.5%) |
| No vomiting or diarrhea | 70 (36%) | 125 (64%) |
